# Affective and Cognitive Vulnerability Under Chronic Stress: Insights From Patients With Left Temporal Lobe Epilepsy and Caregivers

**DOI:** 10.62641/aep.v54i1.2091

**Published:** 2026-02-15

**Authors:** Teresa Vicente-Hernández, Irene Cano-López, Judit Catalán-Aguilar, Paula Tormos-Pons, Kevin G. Hampel, Raquel Ferrer-Ricart, Esperanza González-Bono, Vicente Villanueva

**Affiliations:** ^1^Institut d'Investigació en Psicologia dels Recursos Humans, del Desenvolupament Organitzacional i de la Qualitat de Vida Laboral (IDOCAL)/Department of Psychobiology, Psychology Center, Universitat de València, 46010 Valencia, Spain; ^2^Faculty of Health Sciences, Valencian International University, 46002 Valencia, Spain; ^3^Refractory Epilepsy Unit, Neurology Service, Member of ERN EPICARE, Hospital Universitario y Politécnico La Fe, 46026 Valencia, Spain

**Keywords:** epilepsy, cognition, memory, affective symptoms, caregivers, stress

## Abstract

**Background:**

Temporal lobe epilepsy (TLE) is a chronic stress condition characterized by affective and cognitive deficits. This study analyzed differences in affective and cognitive functioning between patients with TLE and another chronically stressed population — caregivers of patients with epilepsy — as well as the relationships between affective and cognitive outcomes.

**Methods:**

In this cross-sectional study, 40 adults (20 with left TLE and 20 caregivers; mean age 48.43 ± 8.86 years) underwent a neuropsychological assessment evaluating affectivity, attention, executive function, language, and memory.

**Results:**

Patients with TLE and caregivers did not differ in anxiety, depression, attention, executive functions, or visual memory. However, patients with TLE had poorer semantic verbal fluency (*p* = 0.02), naming (*p* < 0.0001), short-term verbal recall (*p* = 0.027), long-term verbal recall with semantic cues (*p* = 0.005), long-term verbal recognition (*p* = 0.017), and verbal discriminability (*p* = 0.001). The group (epilepsy vs. caregiver) significantly moderated the association between depression and long-term verbal recognition (B = –0.12, standard error (SE) = 0.05, *p* = 0.03, 95% confidence interval (CI) [–0.23, –0.01]), with higher depression scores being associated with poorer verbal recognition in patients with epilepsy (*p* = 0.001) but not in caregivers (*p* = 0.74).

**Conclusions:**

These findings suggest a specific pattern of verbal dysfunction and increased cognitive vulnerability to depression in patients with TLE, compared to another chronically stressed group. Although the present study cannot determine the mechanisms underlying these associations, the results underscore the clinical relevance of assessing these variables together and may inform the development of tailored interventions.

## Introduction

Drug-resistant epilepsy can be considered a chronic stress model due to the exposure to recurrent, unpredictable, and uncontrollable seizures, which have cognitive and emotional consequences that affect quality of life [1]. The concept of “allostatic load” provides a valuable framework for understanding how chronic stress exerts cumulative strain on the neuroendocrine system, thereby increasing vulnerability to disease [2]. This concept is particularly relevant in temporal lobe epilepsy (TLE), the most common type of drug-resistant epilepsy, where seizures originate in limbic structures—regions rich in glucocorticoid receptors that are critical to stress regulation, emotional processing, and cognition [3]. In fact, hypothalamic-pituitary-adrenal (HPA) axis dysfunction and basal hypercortisolism have been observed in patients with TLE [4]. Although no direct link has been found between hypercortisolism and negative affectivity in this population [4], hypercortisolism has been related to memory deficits, with clinical variables modulating the affective and cognitive profile of these patients [4, 5].

Although the emotional and cognitive deficits in patients with TLE are well documented [5, 6, 7], it remains unclear to what extent these alterations result from the underlying neurological disease or from chronic exposure to stress. One way to address this question is by comparing patients with epilepsy to another population similarly exposed to chronic stress, but without neurological diseases. Informal caregivers of individuals with epilepsy offer a well-established model of chronic stress, given the substantial emotional and physical demands they face, as well as the unpredictability associated with their relative’s unexpected seizures, which together contribute to chronic stress markers such as a diminished negative feedback mechanism of the HPA axis and elevated cortisol levels [8, 9]. It should be noted that chronic stress in patients with epilepsy additionally involves disease-specific neurobiological processes, including seizure-related network disruption and hippocampal vulnerability [1, 3]. For this reason, caregiver stress is not neurobiologically equivalent to the stress experienced by patients, but caregivers remain an ecologically valid comparison group for isolating the effects of long-term and unpredictable stress in the absence of neurological disease [8, 10, 11]. While previous research has assessed negative affectivity in both patients and caregivers using self-report questionnaires [12], and how patients’ symptoms impact caregiver burden [13], to our knowledge, no research has directly compared their affective and cognitive functioning using comprehensive neuropsychological evaluations. Such a comparison could offer valuable insights into the specific effects of epilepsy versus the broader effects of chronic stress, providing a more nuanced understanding of the mechanisms underlying emotional and cognitive alterations observed in TLE.

This study aims to compare the affective and cognitive profiles of patients with TLE and informal caregivers of individuals with epilepsy, and to examine the relationships between affectivity and cognition. We will also consider the influence of seizure-related clinical variables and caregiver burden. To maximize sample homogeneity and reduce the clinical and neuropsychological variability associated with seizure focus lateralization, only patients with left TLE will be included. Left TLE shows a well-established and consistent pattern of verbal deficits, whereas the association between right TLE and nonverbal impairments is considerably less reliable [14]. Therefore, including patients with right or bilateral TLE would introduce greater cognitive heterogeneity and reduce the interpretability of the findings. We hypothesize that patients with TLE will exhibit higher levels of negative affectivity and poorer cognitive performance compared to caregivers, and that the group (epilepsy vs. caregiver) will moderate the relationship between affectivity and cognitive functioning.

## Materials and Methods

### Participants

Participants were recruited from the Hospital, a center belonging to the European Reference Network for Epilepsy (EpiCARE), between September 2022 and December 2024. Inclusion criteria for the experimental group were: (a) a diagnosis of drug-resistant left TLE; (b) aged 18 years or older; and (c) eligibility for epilepsy surgery to ensure relative homogeneity of the sample. Inclusion criteria for the control group were: (a) being the primary informal caregiver of a patient with drug-resistant epilepsy; (b) aged 18 years or older; and (c) living with the patient. Exclusion criteria for both groups were: (a) presence of neurological, psychiatric, or endocrine diseases (for the experimental group, this criterion refers to diseases different from epilepsy); (b) not having completed primary education; (c) age over 65 years; and (d) lack of fluency in Spanish.

### Procedure

This cross-sectional study was conducted in accordance with the Strengthening the Reporting of Observational Studies in Epidemiology guidelines [15]. The study protocol also adhered to the principles of the Declaration of Helsinki and was approved by the Ethics Committee of the Hospital. All the participants provided informed consent.

For patients with epilepsy, a comprehensive presurgical evaluation was carried out by a multidisciplinary team, which included the assessment of seizure history and semiology, neurological examination, long-term video-electroencephalography (EEG) monitoring, 3-Tesla magnetic resonance imaging (MRI), psychiatric evaluation, and neuropsychological testing for all patients. Additional diagnostic procedures, such as fluorodeoxyglucose-positron emission tomography, single-photon emission computed tomography, and intracranial EEG, were performed selectively. This evaluation enabled accurate classification of the epilepsy type and identification of the lateralization of the epileptogenic focus. Clinical information was recorded. The antiseizure medication (ASM) drug load was calculated using the defined daily dose (DDD), which represents the “assumed average maintenance dose per day” according to the ATC index [16]. For each patient, the daily dose of each ASM was divided by its respective DDD to determine the daily dose-to-DDD ratio. The total drug load from ASMs was then computed by summing the ratios for each ASM prescribed to each patient.

The neuropsychological evaluation was similar for patients with epilepsy and caregivers. Demographic information was collected, including age, gender, education level, marital status, and tobacco use. Handedness was assessed using the Edinburgh Handedness Inventory [17], which determines the dominant hand used for various daily activities. A laterality index ranging from –100 (totally left-handed) to +100 (totally right-handed) was calculated, and individuals were classified as right-handed, left-handed, or ambidextrous. For caregivers, we recorded hours of caregiving per day and perceived social, work, and economic limitations associated with caregiving, evaluated using an ad hoc Likert-type scale ranging from 0 (“none”) to 10 (“very much”), since single-item Likert-type measures have been supported as feasible and valid tools for capturing global subjective constructs burden [18].

### Neuropsychological Assessment

Trait anxiety subscale of the State-Trait Anxiety Inventory (STAI-R), in its Spanish version [19] (Cronbach’s alpha: 0.90). It was used to assess the stable predisposition to experience anxiety. It consists of 20 items rated on a four-point scale. A total direct score was computed (range: 0–60 points), with higher scores indicating higher trait anxiety.

Beck Depression Inventory-II (BDI-II), in its Spanish version [20] (Cronbach’s alpha: 0.87). It was used to assess the severity of depressive symptoms with 21 items rated on a four-point scale (total direct score ranged from 0 to 63), with higher scores indicating higher depression levels.

EpiTrack [21] (Cronbach’s alpha: 0.75). This 15-minute screening tool includes six subtests requiring attention, working memory, and cognitive tracking. A total direct score (range: 9–42 points) was obtained and transformed to an age-adjusted total score (range: 9–49 points), with higher scores indicating better performance.

The Trail Making Test (TMT) A and B [22]. It assesses processing speed, set-shifting, planning, working memory, motor coordination, and visuospatial processing. Part A involves connecting 25 numbered circles (Cronbach’s alpha: 0.79), and part B requires alternating between numbers and letters (Cronbach’s alpha: 0.89). Completion time (seconds) was recorded, with longer times indicating poorer performance.

Iowa Gambling Task (IGT), in its computerized version [23] (Cronbach’s alpha: 0.67). It assesses decision-making under uncertainty. Participants completed 100 trials choosing from decks with different risk–reward profiles. An overall direct score (GI) was calculated by subtracting disadvantageous from advantageous choices (range: –100 to +100).

Tower of London (TOL) (Cronbach’s alpha: 0.68), administered via the PEBL platform [24]. It assesses planning and problem-solving abilities. Participants reproduced a target token configuration by following movement rules. A total direct score (range: 0–36 points) was calculated, with higher scores reflecting better performance.

Stroop Test [25] (Cronbach’s alpha: 0.92). It evaluates inhibitory control and attentional regulation under interference. It includes three tasks: reading color words in black ink, naming the color of X’s, and naming the ink color of incongruent color words. We obtained the number of items correctly indicated in each condition: P (word reading), C (color naming), and PC (color–word interference). An expected interference score (PC^′^) was then calculated using the formula PC^′^ = (P × C) / (P + C), and the final interference index corresponded to PC – PC^′^, with higher positive values reflecting greater difficulty inhibiting automatic reading responses. Because this index is derived from the difference between an observed and an expected performance value, it does not have a fixed theoretical range and can yield both positive and negative scores depending on individual performance.

Wisconsin Card Sorting Test (WCST) [26] (Cronbach’s alpha: 0.92). It assesses cognitive flexibility, abstract reasoning, and feedback-based learning. Participants matched response cards to reference cards based on unstated sorting rules that changed after ten correct trials, requiring strategy shifts. The number of perseverative errors (range: 0–128) was recorded, with higher direct scores reflecting poorer performance.

Verbal Fluency Test (FAS) [27] (Cronbach’s alpha: 0.83). It evaluates phonemic verbal fluency. Participants were asked to generate as many words as possible beginning with the letters F, A, and S within one minute. The total direct score was computed as the sum of all admissible words for the three letters (no fixed maximum score).

Animal Naming Test [28] (Cronbach’s alpha: 0.56). It assesses semantic verbal fluency. Participants were asked to name as many animals as possible in one minute. The total direct score was calculated as the sum of all admissible words for this category (no fixed maximum score).

Boston Naming Test (BNT) [29] (Cronbach’s alpha: 0.86). It evaluates visual confrontation naming. The test consists of 60 graphic stimuli, and participants were instructed to name the represented object. The total direct score was computed as the number of cards correctly named without phonemic cues (range: 0–60 points).

Spanish-Complutense Verbal Learning Test (TAVEC) [30] (Cronbach’s alpha: 0.77–0.86). It assesses episodic verbal memory. List A was presented over five trials to evaluate immediate recall (range: 0–75 points), followed by a single trial of List B. Short-term recall of List A was then assessed, both with and without semantic cues (range: 0–15). After a 20-minute delay, long-term recall with and without semantic cues (range: 0–15), recognition (range: 0–15), and discriminability (range: 0–100) were evaluated. All indices were expressed in direct scores.

The Rey-Osterrieth Complex Figure (ROCF) [31]. It evaluates visuospatial construction and visual memory via copy (Cronbach’s alpha: 0.79) and immediate recall tasks (Cronbach’s alpha: 0.83). The scores were computed as the sum of the drawn elements, considering the degree of accuracy, deformation, and location. According to this correction system, each of the 18 elements of the figure received a score of 0, 0.5, 1, or 2 points. Total direct score ranged from 0 to 36 points, with higher values indicating better performance.

### Statistical Analyses

First, the Shapiro-Wilk test was used to examine data normality. For continuous variables with normal distribution, *t*-tests for independent samples were applied to compare sociodemographic characteristics between groups (patients with epilepsy vs. caregivers). Mann-Whitney U tests were applied when the assumption of normality was not met. Chi-square tests were used to analyze differences in proportions for categorical variables. To explore differences between groups in affective and cognitive variables, *t*-tests for independent samples or Mann-Whitney U tests were employed, as appropriate. Analyses with cognitive variables were repeated, controlling for educational level.

Moderation analyses were performed to explore the possible moderating role of the group (i.e., epilepsy vs. caregivers) in the relationship between affectivity and the cognitive variables in which differences between groups were detected. The PROCESS macro (v. 4; Guilford Press, NY, USA) [32] was used for this purpose, employing a 95% bootstrap confidence interval with bias correction (10,000 iterations). Using the Johnson and Neyman [33] technique, we identified whether the predictor significantly predicted the criterion for each value of the moderator. The simple slopes for each group were graphically represented to estimate the relationship between affectivity and cognitive functioning. We used the computer tool developed by Preacher *et al*. [34] for this analysis. Moderation analyses were repeated, adjusting for structural lesions and educational level.

Spearman correlations were applied to examine the associations among clinical factors, caregiving variables, and affective and cognitive scores. Furthermore, in the group of patients with epilepsy, we explored whether there were differences in affective and cognitive variables based on the presence of brain lesions in MRI using *t*-tests for independent samples and Mann–Whitney U tests, as appropriate.

Multiple testing correction controlling the False Discovery Rate (FDR) was applied in *t*-test for independent samples, correlations, and moderation analyses [35]. The FDR was set to 0.10, which implies that the proportion of significant associations that are actually false discoveries is limited to no more than 10%.

Statistical data analysis was performed using IBM SPSS Statistics software (version 29.0; IBM Corp., Armonk, NY, USA) with *p*-values of 0.05 or less considered significant.

## Results

### Preliminary Analyses

The sample consisted of 40 adults, including 20 patients with drug-resistant left TLE (10 women and 10 men; mean age = 47.70 ± 8.71 years) and 20 caregivers of patients with drug-resistant TLE (10 women and 10 men; mean age = 49.15 ± 9.16 years). Table 1 summarizes the characteristics of both groups.

**Table 1.** **Characteristics of the total sample and groups (mean ± SD or n [%])**.

		Total sample	Epilepsy group (N = 20)	Caregiver group (N = 20)	Statistics
Age (years)		48.43 ± 8.86	47.70 ± 8.71	49.15 ± 9.16	*t*(38) = 0.52, *p* = 0.61
Gender					χ²(1) = 0.00, *p* = 1.00
	Women	20 (50.0%)	10 (50.0%)	10 (50.0%)	
	Men	20 (50.0%)	10 (50.0%)	10 (50.0%)	
Level of education					*t*(38) = –0.37, *p* = 0.97
	Primary	10 (25.0%)	5 (25.0%)	5 (25.0%)	
	Secondary	2 (5.0%)	0 (0.0%)	2 (10.0%)	
	Lower university	12 (30.0%)	6 (30.0%)	6 (30.0%)	
	University	16 (40.0%)	9 (45.0%)	7 (35.0%)	
Handedness					χ²(2) = 1.81, *p* = 0.40
	Right-handed	32 (82.1%)	14 (73.7%)	18 (90.0%)	
	Left-handed	4 (10.3%)	3 (15.8%)	1 (5.0%)	
	Ambidextrous	3 (7.7%)	2 (10.5%)	1 (5.0%)	
Marital status					χ²(2) = 0.15, *p* = 0.93
	Single	9 (22.5%)	5 (25.0%)	4 (20.0%)	
	Married	25 (62.5%)	12 (60.0%)	13 (65.0%)	
	Divorced	6 (15.0%)	3 (15.0%)	3 (15.0%)	
Tobacco use					χ²(1) = 2.13, *p* = 0.14
	Yes	10 (25.00%)	7 (35.00%)	3 (15.00%)	
	No	30 (75.00%)	13 (65.00%)	17 (85.00%)	
Sleep disturbance					χ²(1) = 0.17, *p* = 0.68
	Yes	7 (17.5%)	3 (15.0%)	4 (20.0%)	
	No	33 (82.5%)	17 (85.0%)	16 (80.0%)	
Age at epilepsy onset (years)		-	29.85 ± 16.74	-	
Epilepsy duration (years)		-	17.85 ± 15.39	-	
Seizure frequency (per month)		-	3.60 ± 7.20	-	
Seizure type		-		-	
	FPC	-	1 (5.0%)	-	
	FIC	-	2 (10.0%)	-	
	FBTC	-	1 (5.0%)	-	
	FPC + FIC	-	9 (45.0%)	–	
	FIC + FBTC	-	6 (30.0%)	-	
	FPC + FIC + FBTC	-	1 (5.0%)	-	
Number of ASMs		-	2.53 ± 1.12	-	
Total ASM drug load		-	2.81 ± 1.48	-	
Brain lesion (MRI)		-		-	
	Yes	-	9 (45.00%)	-	
	No	-	11 (55.00%)	-	
MRI diagnosis		-		-	
	Hippocampal sclerosis	-	4 (20.00%)	-	
	Frontal cortical dysplasia	-	2 (10.00%)	-	
	Tumor	-	1 (5.00%)	-	
	Cavernoma	-	2 (10.00%)	-	
	Normal	-	11 (55.00%)	-	
Years providing care				12.00 ± 9.22	
Hours of caregiving per day		-	-	5.41 ± 5.57	
Level of social limitation associated with caregiving		-	-	5.43 ± 3.74	
Level of work limitation associated with caregiving		-	-	4.32 ± 4.31	
Level of economic limitation associated with caregiving		-	-	3.85 ± 3.95	

Note. ASMs, antiseizure medications; FBTC, focal-to-bilateral tonic-clonic seizure; FIC, focal impaired consciousness seizure; FPC, focal preserved consciousness seizure; MRI, magnetic resonance imaging.

Patients with epilepsy and caregivers did not differ in sociodemographic characteristics, including age, gender, educational level, handedness, marital status, or tobacco use. Caregiving-related variables did not show a normal distribution.

### Differences Between Patients With Epilepsy and Caregivers in Negative Affectivity and Cognitive Functioning

All variables showed normal distribution except for EpiTrack score, perseverative errors, copy task, long-term verbal recognition and discriminability, and naming.

**Fig. 1.**
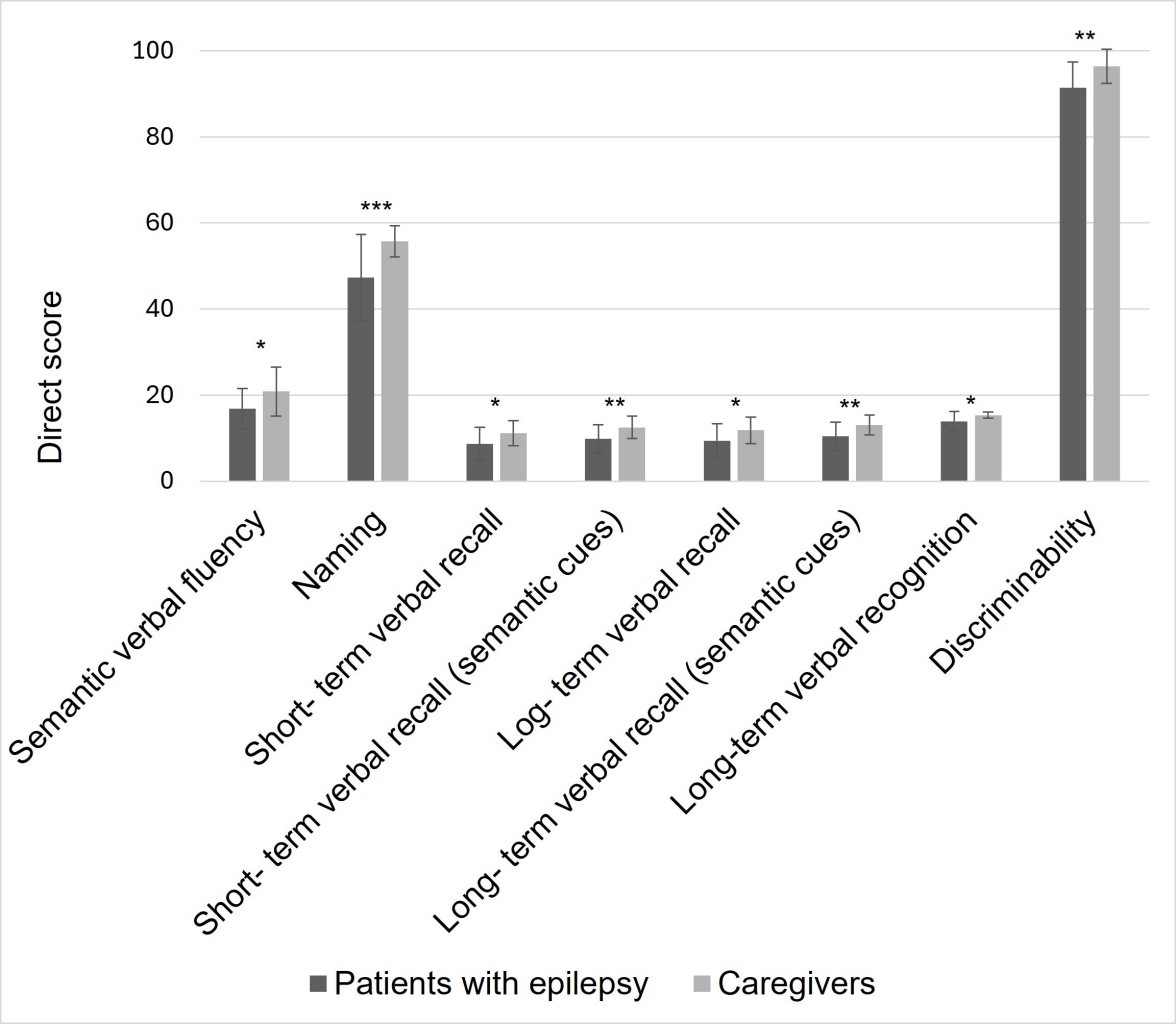
**Language and verbal memory scores in patients with epilepsy and caregivers**. Note: * *p* < 0.05, ** *p* < 0.01, ***p < 0.001.

Significant differences between groups were found in semantic verbal fluency, naming, short-term verbal recall, short-term verbal recall with semantic cues, long-term verbal recall, long-term verbal recall with semantic, long-term verbal recognition and discriminability, with patients with epilepsy having poorer performance than caregivers (Fig. 1). All significant results passed FDR multiple testing correction, except for differences in long-term verbal recall. No significant differences were found in other cognitive scores, trait anxiety, and depression. Table 2 shows statistics for the affective and cognitive variables in each group. These results were maintained even after controlling for educational level.

**Table 2.** **Descriptive statistics for affective and cognitive variables in patients with epilepsy and caregivers (mean ± SD)**.

Variable		Epilepsy group (N = 20)	Caregiver group (N = 20)	Statistics
STAI-R		28.16 ± 11.23	24.55 ± 12.61	*t*(38) = 0.94, *p* = 0.35, *d* = 0.30, 95% CI [–0.33, 0.93]
BDI-II		14.00 ± 9.34	12.70 ± 8.81	*t*(38) = 0.45, *p* = 0.66, *d* = 0.14, 95% CI [–0.49, 0.77]
EpiTrack		31.60 ± 5.98	32.25 ± 4.56	U = 185.00, z = –0.41, *p* = 0.70
TMT A (sec)		33.95 ± 15.87	37.49 ± 13.06	*t*(38) = –0.77, *p* = 0.45, *d* = –0.24, 95% CI [–12.85, 5.76]
TMT B (sec)		84.26 ± 40.37	79.94 ± 34.38	*t*(38) = 0.36, *p* = 0.72, *d* = 0.12, 95% CI [–19.96, 28.61]
IGT		1.44 ± 21.76	3.40 ± 22.84	*t*(38) = –0.27, *p* = 0.79, *d* = –0.09, 95% CI [–16.67, 12.76]
TOL		19.42 ± 6.51	21.45 ± 7.17	*t*(38) = –0.92, *p* = 0.36, *d* = –0.30, 95% CI [–6.48, 2.42]
Interference index (Stroop test)		2.53 ± 5.74	1.76 ± 10.87	*t*(38) = 0.28, *p* = 0.78, *d* = 0.09, 95% CI [–4.79, 6.34]
Perseverative errors (WCST)		14.60 ± 14.58	14.20 ± 14.47	U = 208.50, z = 0.23, *p* = 0.82
FAS		29.75 ± 12.63	38.00 ± 15.60	*t*(38) = –1.84, *p* = 0.07, *d* = –0.58, 95% CI [–1.21, 0.06]
Animal Naming Test		16.85 ± 4.73	20.85 ± 5.68	*t*(38) = –2.42, *p* = 0.02, *d* = –0.77, 95% CI [–1.40, –0.12]*
BNT		47.30 ± 10.06	55.70 ± 3.64	U = 73.00, z = –3.45, *p * < 0.0001***
TAVEC				
	Immediate verbal recall	46.85 ± 11.87	51.05 ± 9.20	*t*(38) = –1.25, *p* = 0.22, *d* = –0.40, 95% CI [–11.00, 2.60]
	Short-term verbal recall	8.65 ± 3.86	11.15 ± 2.94	*t*(38) = –2.31, *p* = 0.027, *d* = –0.73, 95% CI [–1.37, –0.08]*
	Short-term verbal recall with semantic cues	9.80 ± 3.33	12.50 ± 2.63	*t*(38) = –2.85, *p* = 0.007, *d* = –0.90, 95% CI [–1.55, –0.24]**
	Long-term verbal recall	9.40 ± 3.91	11.80 ± 3.05	*t*(38) = –2.16, *p* = 0.037, *d* = –0.68, 95% CI [–1.32, –0.04]*
	Long-term verbal recall with semantic cues	10.40 ± 3.28	13.05 ± 2.26	*t*(38) = –2.97, *p* = 0.005, *d* = –0.94, 95% CI [–1.59, –0.28]**
	Long-term verbal recognition	13.90 ± 2.27	15.35 ± 0.67	U = 112.50, z = –2.54, *p* = 0.017*
	Discriminability	91.41 ± 5.96	96.40 ± 4.02	U = 109.00, z = –2.48, *p* = 0.001**
ROCF				
	Copy	34.48 ± 2.09	34.00 ± 2.73	*t*(38) = 0.62, *p* = 0.64, *d* = 0.20, 95% CI [–1.08, 2.03]
	Immediate visual memory	17.90 ± 5.83	18.66 ± 6.29	*t*(38) = –0.39, *p* = 0.70, *d* = –0.13, 95% CI [–4.69, 3.17]

Note:*p < 0.05, **p < 0.01, ***p < 0.001; BDI-II, Beck Depression Inventory-II; BNT, Boston Naming Test; IGT, Iowa Gambling Task; ROCF, Rey-Osterrieth Complex Figure; STAI-R, trait anxiety subscale of the State-Trait Anxiety Inventory; TAVEC, Spanish-Complutense Verbal Learning Test; TMT, Trail Making Test; TOL, Tower of London; WCST, Wisconsin Card Sorting Test. The interference index (Stroop test) was computed as PC – PC^′^, where PC^′^ = (P × C) / (P + C), based on the number of correctly completed items in each condition (P, C, and PC).

### Relationships Between Negative Affectivity and Cognitive Functioning

**Fig. 2.**
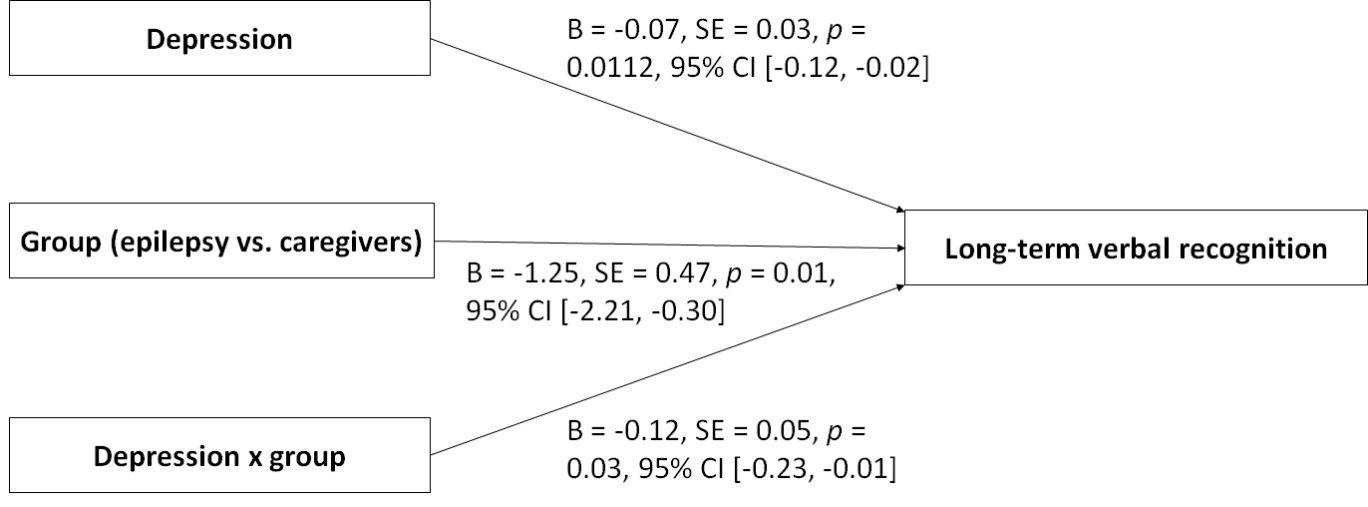
**Model assessing associations of depression, group (epilepsy vs. caregivers), and depression x group with long-term verbal recognition**. Note. CI, confidence interval; SE, standard error.

**Fig. 3.**
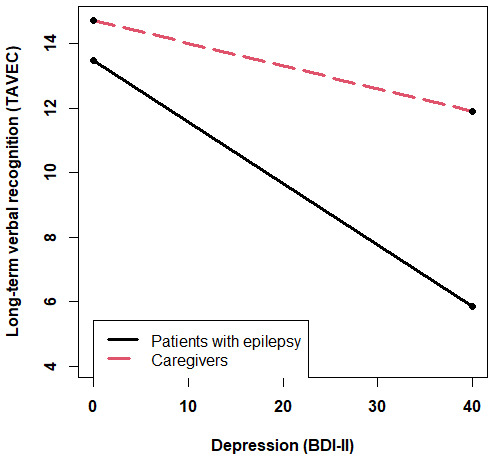
**Association between depression and long-term verbal recognition depending on the group (epilepsy or caregivers)**. Note: BDI-II, Beck Depression Inventory-II; TAVEC, Spanish-Complutense Verbal Learning Test.

In the total sample, correlation analyses revealed a single significant association between depression and immediate visual memory (*r* = –0.39, *p* = 0.015), which did not pass FDR multiple testing correction. No other significant correlations were observed between affective variables and cognitive outcomes. Consequently, we explored whether the group moderated the relationship between affectivity and cognition. Moderation analyses showed that higher depression scores were associated with poorer long-term verbal recognition (B = –0.07, SE = 0.03, *p* = 0.0112, 95% CI [–0.12, –0.02]) (Fig. 2). Additionally, patients with epilepsy showed poorer long-term verbal recognition than caregivers (B = –1.25, SE = 0.47, *p* = 0.01, 95% CI [–2.21, –0.30]) (Fig. 2). The group significantly moderated the association between depression scores and long-term verbal recognition (B = –0.12, SE = 0.05, *p* = 0.03, 95% CI [–0.23, –0.01]) (Fig. 2), with higher depression scores being associated with poorer verbal recognition only in patients with epilepsy (*p* = 0.001) but not in caregivers (*p* = 0.74) (Fig. 3). All significant results passed the FDR multiple testing correction. The model was significant (*F*(3, 35) = 7.10, *p* = 0.0008) and explained 37.8% of the variance in long-term verbal recognition. The moderation effect remained significant after adjusting for structural lesions and educational level. No moderation effects of the group were detected for the relationships between other affective and cognitive variables.

### Relationships of Clinical and Caregiving-Related Variables With Negative Affectivity and Cognitive Functioning

**Fig. 4.**
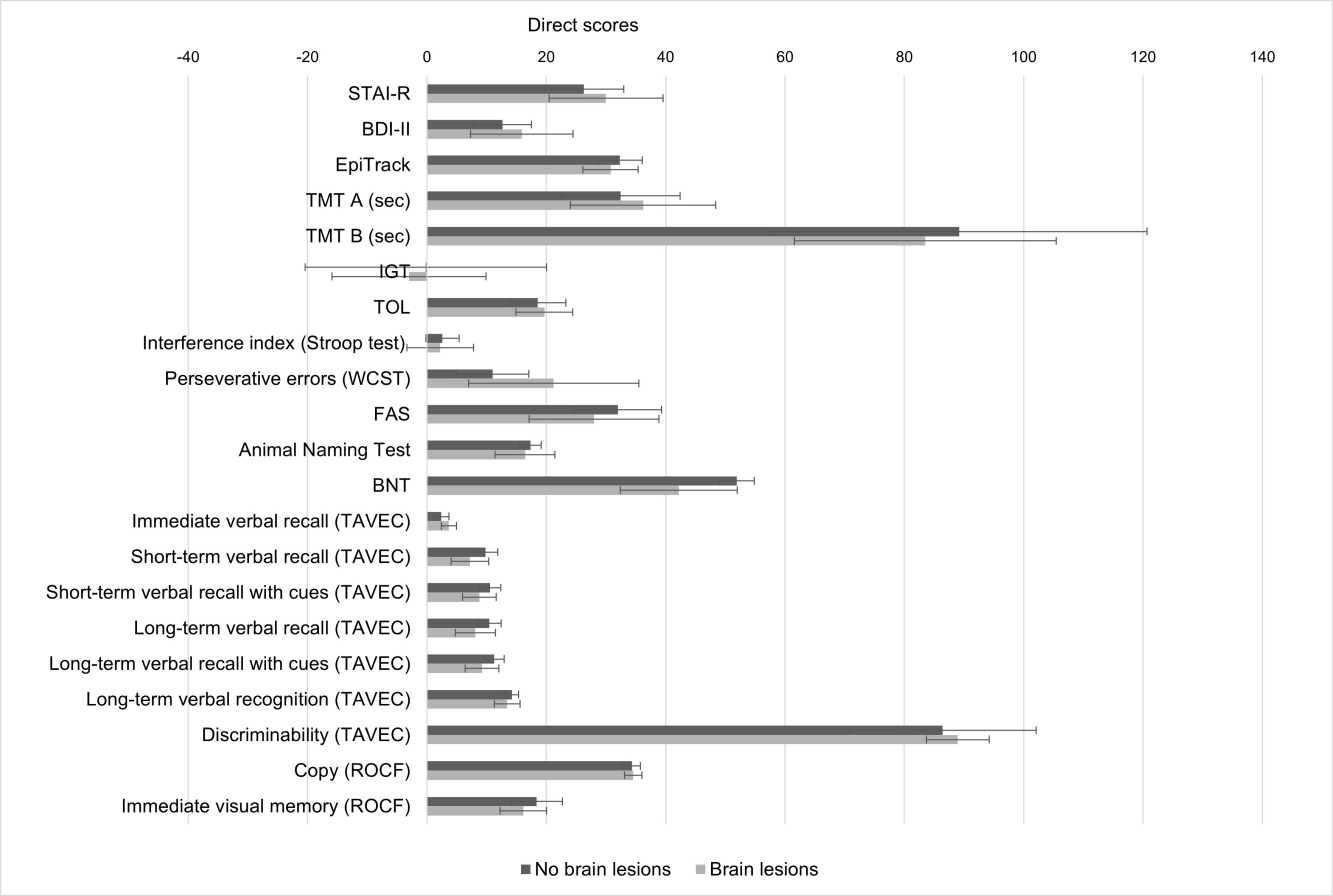
**Mean direct scores on neuropsychological measures in patients with and without brain lesions (as indicated by MRI)**. Note: Error bars represent 95% confidence intervals. Patients with brain lesions showed a tendency toward lower performance on naming (BNT), but differences did not reach statistical significance. BDI-II, Beck Depression Inventory-II; BNT, Boston Naming Test; IGT, Iowa Gambling Task; ROCF, Rey-Osterrieth Complex Figure; STAI-R, trait anxiety subscale of the State-Trait Anxiety Inventory; TAVEC, Spanish-Complutense Verbal Learning Test; TMT, Trail Making Test; TOL, Tower of London.

In patients with epilepsy, no significant correlations were found between clinical variables and affective or cognitive functioning (Table 3). However, patients with brain lesions (as indicated by MRI) had poorer scores on naming than those without brain lesions, although the differences did not reach statistical significance (U = 27.0, z = –1.92, *p* = 0.058) (Fig. 4).

**Table 3.** **Spearman correlations of clinical variables with negative affectivity and cognitive functioning in patients with epilepsy**.

		Age at onset	Epilepsy duration	Seizure frequency	Number of ASMs	Total ASM load
STAI-R		*r* = –0.34, *p* = 0.16	*r* = 0.38, *p* = 0.11	*r* = 0.32, *p* = 0.19	*r* = 0.26, *p* = 0.30	*r* = 0.18, *p* = 0.50
BDI-II		*r* = –0.24, *p* = 0.33	*r* = 0.28, *p* = 0.25	*r* = 0.35, *p* = 0.15	*r* = 0.12, *p* = 0.63	*r* = 0.01, *p* = 0.99
EpiTrack		*r* = –0.05, *p* = 0.85	*r* = –0.11, *p* = 0.63	*r* = –0.18, *p* = 0.45	*r* = –0.69, *p* = 0.78	*r* = –0.27, *p* = 0.27
TMT A (sec)		*r* = –0.02, *p* = 0.94	*r* = 0.78, *p* = 0.75	*r* = 0.25, *p* = 0.30	*r* = 0.17, *p* = 0.49	*r* = 0.39, *p* = 0.11
TMT B (sec)		*r* = –0.14, *p* = 0.57	*r* = 0.25, *p* = 0.30	*r* = 0.32, *p* = 0.18	*r* = 0.23, *p* = 0.35	*r* = 0.46, *p* = 0.06
IGT		*r* = 0.13, *p* = 0.60	*r* = –0.14, *p* = 0.58	*r* = –0.04, *p* = 0.88	*r* = 0.28, *p* = 0.28	*r* = 0.14, *p* = 0.59
TOL		*r* = 0.20, *p* = 0.40	*r* = –0.29, *p* = 0.23	*r* = –0.15, *p* = 0.53	*r* = 0.12, *p* = 0.65	*r* = –0.08, *p* = 0.76
Interference index (Stroop test)		*r* = –0.15, *p* = 0.54	*r* = 0.16, *p* = 0.49	*r* = –0.32, *p* = 0.17	*r* = 0.02, *p* = 0.93	*r* = 0.01, *p* = 0.97
Perseverative errors (WCST)		*r* = 0.05, *p* = 0.82	*r* = 0.02, *p* = 0.92	*r* = 0.42, *p* = 0.07	*r* = 0.12, *p* = 0.61	*r* = 0.11, *p* = 0.65
FAS		*r* = 0.25, *p* = 0.28	*r* = –0.28, *p* = 0.23	*r* = –0.07, *p* = 0.78	*r* = 0.21, *p* = 0.39	*r* = –0.02, *p* = 0.95
Animal Naming Test		*r* = –0.07, *p* = 0.76	*r* = –0.01, *p* = 0.96	*r* = 0.08, *p* = 0.74	*r* = 0.21, *p* = 0.38	*r* = 0.09, *p* = 0.71
BNT		*r* = 0.14, *p* = 0.56	*r* = –0.15, *p* = 0.53	*r* = –0.16, *p* = 0.49	*r* = 0.05, *p* = 0.85	*r* = –0.00, *p* = 0.99
TAVEC						
	Immediate verbal recall	*r* = 0.32, *p* = 0.17	*r* = –0.31, *p* = 0.19	*r* = 0.04, *p* = 0.86	*r* = 0.19, *p* = 0.43	*r* = 0.05, *p* = 0.84
	Short-term verbal recall	*r* = 0.20, *p* = 0.40	*r* = –0.16, *p* = 0.49	*r* = 0.03, *p* = 0.89	*r* = 0.14, *p* = 0.56	*r* = –0.89, *p* = 0.73
	Short-term verbal recall with cues	*r* = 0.29, *p* = 0.21	*r* = –0.25, *p* = 0.30	*r* = 0.06, *p* = 0.81	*r* = 0.20, *p* = 0.42	*r* = –0.01, *p* = 0.96
	Long-term verbal recall	*r* = 0.21, *p* = 0.37	*r* = –0.16, *p* = 0.49	*r* = –0.12, *p* = 0.62	*r* = 0.04, *p* = 0.87	*r* = 0.14, *p* = 0.58
	Long-term verbal recall with cues	*r* = 0.22, *p* = 0.35	*r* = –0.19, *p* = 0.42	*r* = 0.01, *p* = 0.97	*r* = 0.12, *p* = 0.62	*r* = –0.82, *p* = 0.75
	Long-term verbal recognition	*r* = 0.11, *p* = 0.64	*r* = –0.07, *p* = 0.77	*r* = –0.24, *p* = 0.32	*r* = 0.01, *p* = 0.96	*r* = –0.14, *p* = 0.58
	Discriminability	*r* = 0.22, *p* = 0.36	*r* = –0.17, *p* = 0.48	*r* = 0.04, *p* = 0.87	*r* = 0.20, *p* = 0.42	*r* = 0.08, *p* = 0.75
ROCF						
	Copy	*r* = –0.02, *p* = 0.94	*r* = –0.00, *p* = 0.99	*r* = –0.24, *p* = 0.30	*r* = 0.00, *p* = 1.00	*r* = 0.01, *p* = 0.98
	Immediate visual memory	*r* = –0.04, *p* = 0.88	*r* = –0.02, *p* = 0.94	*r* = –0.27, *p* = 0.25	*r* = 0.28, *p* = 0.24	*r* = 0.14, *p* = 0.59

Note: BDI-II, Beck Depression Inventory-II; BNT, Boston Naming Test; IGT, Iowa Gambling Task; ROCF, Rey-Osterrieth Complex Figure; STAI-R, trait anxiety subscale of the State-Trait Anxiety Inventory; TAVEC, Spanish-Complutense Verbal Learning Test; TMT, Trail Making Test; TOL, Tower of London; WCST, Wisconsin Card Sorting Test; FAS, FAS Verbal Fluency Test.

In caregivers (Table 4), depression scores tended to be related to higher perceived social limitations associated with caregiving. Semantic verbal fluency was negatively associated with daily hours of caregiving, social limitations, and work limitations. Additionally, phonemic verbal fluency was negatively associated with economic limitations. Only the relationship between semantic verbal fluency and daily hours of caregiving passed FDR multiple testing correction. No other significant correlations were found.

**Table 4.** **Spearman correlations of caregiving-related variables with negative affectivity and cognitive functioning in the group of caregivers**.

		Hours of caregiving per day	Level of social limitation	Level of work limitation	Level of economic limitation
STAI-R		*r* = 0.18, *p* = 0.45	*r* = 0.36, *p* = 0.12	*r* = 0.34, *p* = 0.14	*r* = –0.03, *p* = 0.89
BDI-II		*r* = 0.24, *p* = 0.30	*r* = 0.42, *p* = 0.068	*r* = 0.39, *p* = 0.09	*r* = 0.04, *p* = 0.86
EpiTrack		*r* = –0.02, *p* = 0.42	*r* = –0.25, *p* = 0.28	*r* = –0.29, *p* = 0.22	*r* = –0.39, *p* = 0.09
TMT A (sec)		*r* = 0.16, *p* = 0.50	*r* = –0.01, *p* = 0.96	*r* = 0.05, *p* = 0.83	*r* = 0.03, *p* = 0.91
TMT B (sec)		*r* = –0.11, *p* = 0.63	*r* = –0.02, *p* = 0.95	*r* = 0.09, *p* = 0.71	*r* = –0.02, *p* = 0.93
IGT		*r* = –0.33, *p* = 0.16	*r* = –0.26, *p* = 0.28	*r* = –0.12, *p* = 0.61	*r* = –0.22, *p* = 0.34
TOL		*r* = 0.02, *p* = 0.93	*r* = –0.19, *p* = 0.44	*r* = –0.22, *p* = 0.36	*r* = –0.18, *p* = 0.45
Interference index (Stroop test)		*r =* 0.14, *p* = 0.55	*r* = 0.12, *p* = 0.61	r = 0.19, *p* = 0.43	*r* = –0.02, *p* = 0.93
Perseverative errors (WCST)		*r* = 0.13, *p* = 0.59	*r* = 0.05, *p* = 0.82	*r* = –0.09, *p* = 0.71	*r* = 0.15, *p* = 0.53
FAS		*r* = –0.14, *p* = 0.57	*r* = –0.24, *p* = 0.31	*r* = –0.33, *p* = 0.16	*r* = –0.47, *p* = 0.038*
Animal Naming Test		*r* = –0.56, *p* = 0.01*	*r* = –0.61, *p* = 0.005*	*r* = –0.70, *p* = 0.001*	*r* = –0.37, *p* = 0.10
BNT		*r* = 0.05, *p* = 0.84	*r* = –0.04, *p* = 0.86	*r* = –0.22, *p* = 0.36	*r* = –0.21, *p* = 0.38
TAVEC					
	Immediate verbal recall	*r* = –0.03, *p* = 0.91	*r* = –0.14, *p* = 0.55	*r* = –0.18, *p* = 0.45	*r* = –0.03, *p* = 0.89
	Short-term verbal recall	*r* = 0.01, *p* = 0.97	*r* = –0.11, *p* = 0.65	*r* = 0.03, *p* = 0.89	*r* = –0.14, *p* = 0.54
	Short-term verbal recall with semantic cues	*r* = 0.09, *p* = 0.69	*r* = –0.09, *p* = 0.71	*r* = –0.17, *p* = 0.46	*r* = –0.11, *p* = 0.64
	Long-term verbal recall	*r* = 0.16, *p* = 0.50	*r* = –0.01, *p* = 0.98	*r* = 0.10, *p* = 0.68	*r* = –0.06, *p* = 0.80
	Long-term verbal recall with semantic cues	*r* = 0.10, *p* = 0.67	*r* = –0.08, *p* = 0.74	*r* = –0.01, *p* = 0.97	*r* = –0.15, *p* = 0.51
	Long-term verbal recognition	*r* = 0.13, *p* = 0.59	*r* = 0.03, *p* = 0.92	*r* = 0.17, *p* = 0.47	*r* = –0.30, *p* = 0.91
	Discriminability	*r* = 0.48, *p* = 0.031*	*r* = 0.38, *p* = 0.10	*r* = 0.19, *p* = 0.42	*r* = 0.29, *p* = 0.22
ROCF					
	Copy	*r* = –0.10, *p* = 0.75	*r* = –0.30, *p* = 0.19	*r* = –0.06, *p* = 0.79	*r* = –0.33, *p* = 0.16
	Immediate visual memory	*r* = –0.14, *p* = 0.57	*r* = –0.47, *p* = 0.042*	*r* = –0.41, *p* = 0.08	*r* = –0.11, *p* = 0.65

Note: *: *p* < 0.05. BDI-II, Beck Depression Inventory-II; BNT, Boston Naming Test; IGT, Iowa Gambling Task; ROCF, Rey-Osterrieth Complex Figure; STAI-R, trait anxiety subscale of the State-Trait Anxiety Inventory; TAVEC, Spanish-Complutense Verbal Learning Test; TMT, Trail Making Test; TOL, Tower of London; WCST, Wisconsin Card Sorting Test; FAS, FAS Verbal Fluency Test.

## Discussion

This study examined differences in affective and cognitive functioning between patients with left TLE and another chronically stressed population — caregivers of patients with epilepsy. Although both groups showed similar levels of negative affectivity and comparable performance in attention, executive functions, and visual memory, patients with epilepsy exhibited significantly poorer performance in verbal domains (fluency, naming, and memory). Additionally, the group (epilepsy vs. caregivers) moderated the relationship between depression and verbal memory, with depression scores being associated with poorer verbal memory in patients with epilepsy, but not in caregivers.

Results did not reveal significant differences in negative affectivity between patients with epilepsy and caregivers, contrary to our hypothesis. Although individuals with TLE face distinctive challenges — such as recurrent exposition to unpredictable and uncontrollable seizures and underlying neurological dysfunction — our findings suggest that the chronic and unpredictable nature of epilepsy exerts a comparable psychological impact on caregivers. Caregivers are frequently exposed to emotional stress [13]. Tsamakis *et al*. [12] reported high levels of anxiety and depression in both patients and caregivers, highlighting a shared affective burden. Our findings suggest that negative affectivity may be more related to chronic stress from living with a chronic illness or being a caregiver than to the presence of neurological damage.

As hypothesized, our results showed that patients with epilepsy showed poorer performance than caregivers in verbal-related functions (i.e., verbal fluency, naming, and verbal memory), with no differences emerged in nonverbal domains, even after controlling for educational level, and this selective impairment in tasks mainly mediated by left temporal-mesial structures (e.g., the hippocampus) aligns with the material-specificity hypothesis, which posits left-hemisphere specialization for verbal memory and right-hemisphere specialization for nonverbal memory [14, 36]. Supporting this view, recent studies have shown that left temporal lobe dysfunction is associated with selective impairments in temporal-linguistic functions such as lexical retrieval, verbal memory, and fluency [32, 37].

We found that depressive symptoms were significantly associated with poorer long-term verbal recognition in patients with epilepsy, but not in caregivers, and this pattern suggests that the impact of negative affectivity on cognition may be specific to the clinical group [38, 39]. Although the present study cannot directly assess underlying neural mechanisms, prior evidence indicates that patients with left temporal lobe dysfunction may be especially vulnerable to the cognitive effects of depression, likely reflecting the role of the temporal lobe in both seizure activity and the regulation of emotion and cognition [1, 3]. Consistently, Helmstaedter *et al*. [40] emphasized the role of left-hemispheric language networks in mediating the relationship between depression and verbal memory deficits. Structural changes, such as reduced left hippocampal volume — frequently reported in both TLE and depressive disorders — may heighten susceptibility to depression-related cognitive dysfunction [41]. Neurochemical mechanisms may also contribute; for example, decreased 5-HT1A receptor binding in the left hippocampus has been linked to depressive symptoms and poorer delayed verbal memory [42]. In contrast, despite comparable levels of depression, caregivers did not exhibit this association, probably due to the relatively preserved structural integrity of frontotemporal and limbic networks.

In our study, most clinical variables examined in the epilepsy group were not significantly associated with affective or cognitive functioning, but, given the relatively small sample size, these null correlations should be interpreted with caution, as limited statistical power may have prevented the detection of subtle associations [43]. In this context, we found a trend toward poorer naming performance in patients with structural brain lesions. These findings underscore the importance of considering structural and functional neuroanatomy when evaluating cognitive and affective outcomes in epilepsy. In the caregiver group, our findings revealed a significant association between daily hours of caregiving and poorer semantic verbal fluency. These findings support the view that sustained caregiving burden is a psychological risk factor for cognitive vulnerability.

A strength of this study is that, to our knowledge, it is the first to compare affective and cognitive functioning in patients with drug-resistant left TLE and caregivers of individuals with epilepsy — two populations exposed to chronic stress — using a comprehensive neuropsychological assessment. By assessing a wide range of cognitive and emotional domains, the study offers valuable implications for designing targeted interventions for both groups. Methodologically, sample homogeneity was enhanced by restricting inclusion to patients with left TLE, thereby reducing variability associated with the side of seizure focus. Despite these strengths, some limitations should be noted. First, the sample was relatively small, so larger samples would increase statistical power, allowing for a more reliable examination of clinical predictors. Second, the cross-sectional design of the study prevents the establishment of causal relationships between variables; longitudinal studies are needed to explore temporal dynamics and directionality in the relationship between affective and cognitive variables. Third, although standardized neuropsychological tests were used, future research could benefit from functional neuroimaging techniques to better understand the brain mechanisms underlying the relationship between affectivity and cognitive functioning. Fourth, anxiety and depression scores are not clinical measures (since we considered anxiety as a trait, and depression was only measured once), and so this does not enable establishing a diagnostic criterion. Fifth, although caregivers provide an ecologically comparable control group, potential confounding factors should be considered, as pre-existing differences in baseline cognitive abilities could influence group comparisons. Future studies should include an additional control group of healthy participants to better account for these potential factors. Finally, because moderation analyses typically require larger samples and interaction effects are difficult to detect, results should be interpreted with caution. Although we used bias-corrected bootstrap confidence intervals, which are particularly useful when sampling distributions are unknown or unstable [32], the limited sample size reduces statistical power.

## Conclusions

Our findings highlight the complex interplay between emotional and cognitive functioning in patients with TLE and caregivers. Although both groups exhibited comparable affective profiles and similar performance on nonverbal cognitive tasks, a distinct dissociation emerged in verbal domains, with patients showing greater impairment. Notably, depression scores were linked to poorer delayed verbal memory exclusively in the epilepsy group. These results may have clinical implications, underscoring the need for comprehensive, group-specific interventions that consider both affective and cognitive dimensions.

## Availability of Data and Materials

The data that support the findings of this study are available from the corresponding author, upon request.
